# Deciphering structure and topology of conserved COG2042 orphan proteins

**DOI:** 10.1186/1472-6807-5-3

**Published:** 2005-02-08

**Authors:** Jean Armengaud, Alain Dedieu, Olivier Solques, Jean-Luc Pellequer, Eric Quemeneur

**Affiliations:** 1 CEA-VALRHO, DSV-DIEP-SBTN, Service de Biochimie post-génomique & Toxicologie Nucléaire, Bagnols-sur-Cèze, France

## Abstract

**Background:**

The cluster of orthologous group COG2042 has members in all sequenced Eukaryota as well as in many Archaea. The cellular function of these proteins of ancient origin remains unknown. PSI-BLAST analysis does not indicate a possible link with even remotely-related proteins that have been functionally or structurally characterized. As a prototype among COG2042 orthologs, SSO0551 protein from the hyperthermophilic archaeon *Sulfolobus solfataricus *was purified to homogeneity for biophysical characterization.

**Results:**

The untagged protein is thermostable and behaves as a monomeric protein in gel filtration experiment. Several mass spectrometry-based strategies were combined to obtain a set of low resolution structural information. Kinetic data from limited proteolysis with various endoproteases are concordant in pointing out that region Glu^73^-Arg^78 ^is hyper-sensitive, and thus accessible and flexible. Lysine labeling with NHS-biotin and cross-linking with DTSSP revealed that the 35 amino acid RLI motif at the N terminus is solvent exposed. Cross-links between Lys^10^-Lys^14 ^and Lys^23^-Lys^25 ^indicate that these residues are spatially close and in adequate conformation to be cross-linked. These experimental data have been used to rank multiple three-dimensional models generated by a *de novo *procedure.

**Conclusion:**

Our data indicate that COG2042 proteins may share a novel fold. Combining biophysical, mass-spectrometry data and molecular model is a useful strategy to obtain structural information and to help in prioritizing targets in structural genomics programs.

## Background

Genomic comparative studies on entirely sequenced genomes from the three domains of life, i.e. Bacteria, Archaea and Eukaryota [1], evidenced that proteins involved in the organization or processing of genetic information (structures of ribosome and chromatin, translation, transcription, replication and DNA repair) display a closer relationship between Archaea and Eukaryota than between Bacteria and Eukaryota [2-4]. To identify new proteins involved in such important cellular mechanisms, an exhaustive inventory of proteins of unknown function common to only Eukaryota and Archaea but not in Bacteria has been devised [5-7]. Among such proteins, the Cluster of Orthologous Group COG2042 comprises proteins ubiquitously present in Eukaryota and present in many, but not all, Archaea; a hallmark of their ancient origin. The corresponding ancestral protein should have been present in the common ancestor of these two domains of life. Some partial experimental data are known from the *Saccharomyces cerevisiae *COG2042 homolog. Deletion of the *Yor006c *gene was shown to result in a viable phenotype but some apparent moderate growth defects were noticed on a fermentable carbon source [8,9]. Two putative protein partners for Yor006c were identified through a high-throughput two-hybrid study [10]: Ydl017w, a serine/threonine kinase also known as the cell division control protein 7 (Cdc7), and Yil025c, a hypothetical ORF. However, the cellular function of COG2042 proteins remains unknown.

A polar region, named RLI, is conserved at the N terminus of COG2042 proteins as well as at the N terminus of another cluster of orthologous proteins, namely COG1245. The latter, exemplified by SSO0287 in *Sulfolobus solfataricus *[11], are large proteins (about 600 residues) that encompass four different domains: a RLI domain, a [4Fe-4S] ferredoxin domain, and two ATPase domains, usually found in ABC transporter. Their putative function is currently subjected to discussion [12,13] but could be related to rRNA metabolism. Indeed, four of the eleven proteins shown to interact with the yeast COG1245 homolog (Ydr091c) were identified as involved in rRNA metabolism (Ymr047c, Ydl213c, Ylr340w, Ylr192c). Experimental data on the human homolog of Ydr091c indicated that this protein reversibly associates with RnaseL, and thus COG1245 proteins were named RNase L inhibitor [14].

Because knowledge of protein structure is of high importance to understand protein function, huge efforts have been recently invested in high-throughput protein structure determination programs [15]. Recent reports indicate that only a relatively small percentage of expressed and purified proteins are amenable to full 3D structure by NMR or crystallography and X-ray diffraction [16,17]. *In silico *modeling (homology modeling, fold recognition, *ab initio *and *de novo *modeling) is the alternative to quickly gain the fold of a protein. However, such approach sometimes remains ambiguous in reliably identifying correct structures for protein sequences remotely-related to those found in PDB database. A promising strategy is the use of experimental data (if possible easily obtained) for model discrimination or refinement [18-20]. For example, the tertiary structure of the bovine basic fibroblast growth factor (FGF)-2 was probed with a lysine-specific cross-linking agent and subjected to tryptic peptide mapping by mass spectrometry to identify the sites of cross-linking [21]. The low resolution interatomic distance information obtained experimentally allowed the authors to distinguish among threading models in spite of a relatively low sequence similarity (13 % of identical residues). Interestingly, the constant development of novel cross-linking reagents suitable for mass spectrometry [22] enables enrichment of cross-linked peptides facilitating such strategy. A chemical modification approach [23-26], in combination with limited proteolysis procedures [27,28], can also provide useful structural constraints [29] for model refinement.

A step further is to attempt such approaches with proteins having no detectable homologs. In order to get insight into the topology of COG2042 members and if possible to use these experimental data to discriminate among structural protein templates, we combined limited proteolysis, lysine labeling and cross-linking strategies. The protein SSO0551 from the hyperthermophilic archaea *Sulfolobus solfataricus *was chosen as a prototype because of its thermostability and the probable absence of post-translational modifications when produced as a recombinant form in *Escherichia coli*. The SSO0551 protein is monomeric with a low molecular mass (19 kDa). This size is easily amenable to characterization by mass spectrometry. Our results reveal that the polar RLI motif at the N terminus is probably structured and solvent exposed, pointing at a common trait between COG2042 and COG1245 proteins, this latter group being also conserved in Eukaryota and Archaea but absent in Bacteria. The accessible and flexible regions defined by limited proteolysis combined with lysine accessibility assessed by NHS-biotin labeling and DTSSP cross-linking allowed us to discriminate among ten top ranking *de novo *three-dimensional (3D) models.

## Results

### COG2042 comprises members exclusively from Eukaryota and Archaea

**Figure 1:**
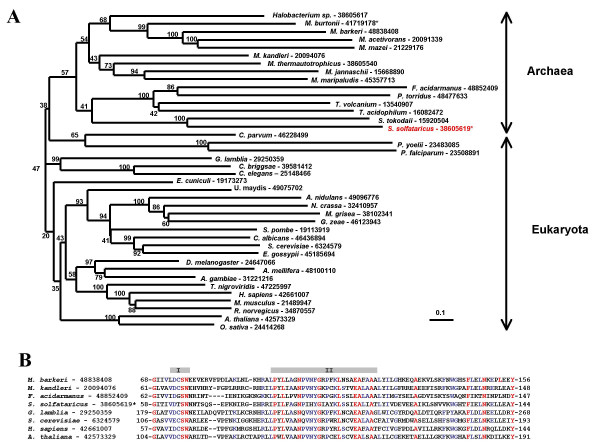
**COG2042 sequences comparison**. A – Phylogenetic analysis of archaeal and eukaryal COG2042 sequences. Archaeal and eukaryal homologs were obtained from public databases [54] by PSI-BLAST searches. To get the most conserved alignment between COG2042 polypeptide sequences, most probable start codons should be considered as atg at nucleotide 500978 on Crick strand for MTH554 from *Methanothermobacter thermoautotrophicus *str. DeltaH (NC_000916), gtg at nucleotide 1526308 on Watson strand for Vng2075c from *Halobacterium *sp. NRC-1 (NC_002607), atg at nucleotide 8398 on Watson strand for Mbur141901 from *Methanococcoides burtonii *DSM6242 (NZ_AADH01000008) and atg at nucleotide 484790 on Crick strand for SSO0551 from *S. solfataricus *(NC_002754). Asterisks indicate modified protein sequences according to this new proposed annotation. Multiple alignments were performed by ClustalW. Following removal of a few ambiguously aligned regions, a data set was assembled comprising 40 sequences over 162 amino acid positions. An unrooted evolutionary distance tree was constructed based on Kimura distances and neighbor joining tree reconstruction algorithm. Bootstrap confidence levels at nodes were computed by the Phylips package with 400 replicates. Scale bar represents unit of amino acid substitutions per position. Accession numbers (gi) are indicated beside the organism. B – Conserved sequence blocks in the alignment of COG2042 members. Based on the phylogenetic analysis (Panel A), eight representative sequences were selected out of 40 COG2042 sequences. Four first sequences are from Archaea while last four sequences are from Eukaryota. SSO0551 sequence (gi38605619) from *S. solfataricus*, labeled with an asterisk, has been numbered according to the new annotation proposed in RESULTS section. Invariant residues in the eight sequences are shown in red and conserved residues in blue. Two conserved motifs commented in the results section are indicated with grey boxes.

The sequence of SSO0551 from *S. solfataricus *was used as query in a PSI-BLAST database search to identify homologous proteins. A constant cutoff expectation value of 10^-15 ^resulted after three iterations in selection of 40 sequences (15 from Archaea and 25 from Eukaryota) that were all aligned over their full length. No close homologs (E-value below 10^-10 ^in the third iteration) with full-length sequence matching to SSO0551 were found among Bacteria. Remarkably, all completely sequenced Eukaryal organisms were found to have one SSO0551 homolog. Fig. 1 shows an unrooted phylogram of the updated COG2042 family (Fig. 1A) and an alignment of a selection of six representative sequences (Fig. 1B), selected on the basis of their phylogenetic distribution. When experimental evidences concerning the protein are unfortunately lacking for ORF description genome annotators usually take into consideration the most upstream initiation codon. For this reason, the most probable start codons of several open reading frames should be reconsidered after exhaustive alignment (Fig. 1). For example, atg codon starting at nucleotide 484790 on the Crick strand for SSO0551 from *S. solfataricus *(NC_002754) should be a more appropriate start codon than atg starting at nucleotide 484916 and mentioned erroneously in current database. From the unrooted phylogram (Fig. 1A), two main lineages (archaeal and eukaryal) can be defined based on organism origin. This suggests that occurrence of these proteins is at least as ancient as divergence of these two phyla. No paralogs, sign of a possible evolution of a new derived function, have been evidenced in entirely sequenced organisms currently available. Although these proteins are of ancient origin, the core sequence appears well conserved as observed in Fig. 1B. Thirty-three residues (38%) are found identical in the core central segment (out of 88 amino acids) between the most distant COG2042 orthologs, namely gi48852409 from *Ferroplasma acidarmanus *and gi6324579 from *Saccharomyces cerevisiae *(Fig. 1B). From the alignment, several conserved motifs that may be functionally crucial (cofactor or substrate binding, catalysis, or partner interactions) were detected. A conserved hexapeptide sequence, Val-Val/Ile/Leu-Asp/Glu-Cys-Ser-Trp (motif I in Fig. 1B), is found distant of 14–17 amino acids from another conserved motif of 25 amino acids containing 4 polar, 18 hydrophobic and 3 aromatic residues (motif II). Database searches with these motifs as queries did not allow identification of remotely-related proteins. All sequences from COG2042 encompass a stretch of 35 conserved amino acids upstream of the core common sequence. This motif, called RLI, is extremely polar (11 basic and 4 acidic residues) and is also found at the N terminus of another group of orthologous sequences, namely COG1245.

### Expression in *E. coli *of two engineered SSO0551 constructs

From multiple sequence alignments, *SSO0551 *should encode a 166 amino acid polypeptide. An N-terminal 6His tagged recombinant construct (pSBTN-AB31) was engineered. As we could not exclude that the 42 amino acids extension at the N terminus was not an annotation artifact, we intended to check experimentally whether this putative extension could have some influence on SSO0551. A second construct (pSBTN-AB30) was simultaneously engineered supposedly allowing production of a 26 kDa N-terminal 6His variant. Unexpectedly, no major difference in expression was detected between the two cellular extracts when they were resolved on SDS-PAGE. Two overexpressed products with both an apparent molecular weight of approximately 20 kDa were obtained upon addition of IPTG (data not shown). Fingerprint identification of these two products was carried out by trypsin proteolysis and mass spectrometry. Table 1 shows the MALDI-TOF mass measurements recorded for the two samples. The tryptic peptides that were detected revealed that both products correspond to native SSO0551 sequence. From the 6His-SSO0551 product (pSBTN-AB31 construct), thirteen peptides map with the theoretical sequence (57 % sequence coverage). Noteworthy, a peptide (1590.64 amu) was attributed to part of the 6His-modified N terminus (Table 1). The twelve peptides recorded from the 6His-SSO0551 extended version (pSBTN-AB30 construct) fit only to the C terminus of the theoretical construct (43 % sequence coverage). These results along with low molecular weight observation on SDS-PAGE indicate that probably a truncated protein was obtained during expression of the ORF comprising the 126 nt 5'-extension (42 additional amino acids at the N-terminus). This product, corresponding in fact to untagged SSO0551 as confirmed hereafter with purified product, showed no binding on Ni-NTA chromatography. This observation is in agreement with absence of 6His tag at the N terminus.

### Recombinant SSO0551 is structured, thermostable and monomeric

**Figure 2:**
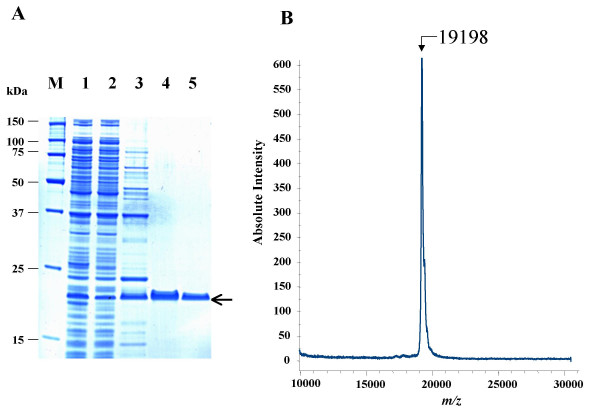
**Analysis of untagged SSO0551 purification**. A – Various purification steps followed by SDS-PAGE. SDS-PAGE was performed on a 4–12% gradient Novex bis-tris acrylamide gel (Invitrogen) and stained with BlueSafe Coomassie solution (Biorad). Lane M, molecular weight markers; Lane 1, cell-free extract of *E. coli *Rosetta(DE3)(pLysS)(pSBTN-AB30) ; Lane 2, soluble proteins from cell-free extract; Lane 3, soluble proteins after the 60°C heat treatment; Lane 4, Resource-S eluate ; Lane 5, Superdex75 eluate. Bands corresponding to recombinant SSO0551 polypeptide are indicated with the arrow. B – SSO0551 mass measurement by MALDI-TOF mass spectrometry. Measurements from 250 laser shots of purified protein cristallized with sinapinic acid were averaged.

Crude extract containing native untagged SSO0551 polypeptide from *E. coli *Rosetta(DE3)(pLysS)(pSBTN-AB30) cells was heated at different temperatures. Proteins that remained soluble were analyzed on SDS-PAGE. Most of *E. coli *contaminants were removed by such treatment. SSO0551 polypeptide remained soluble even when cell extract was heated to 80°C and therefore this protein was considered as thermostable. This protein was purified to homogeneity by a three-step purification protocol. A 20 min heat treatment at 70°C (Fig. 2A, lane 3), followed by a Resource-S ion exchange chromatography (Fig. 2A, lane 4) and a Superdex75 gel filtration (Fig. 2A, lane 5), yielded approximately 1.6 mg of pure protein per L of culture. Purified protein was subjected to MALDI-TOF mass analysis. Fig. 2B shows the spectrum recorded. The experimental *m/z *of 19,198 measured for the monocharged polypeptide matches perfectly with theoretical average mass of native untagged SSO0551 protein (average mass of 19,197 Da). This measurement unequivocally confirmed that a truncated protein is produced using *E. coli *Rosetta(DE3)pLysS transformed with pSBTN-AB30. Both SDS-PAGE (Fig. 2A, lane 5) and MALDI-TOF spectrum (Fig. 2B) testify for homogeneity of the sample.

**Figure 3:**
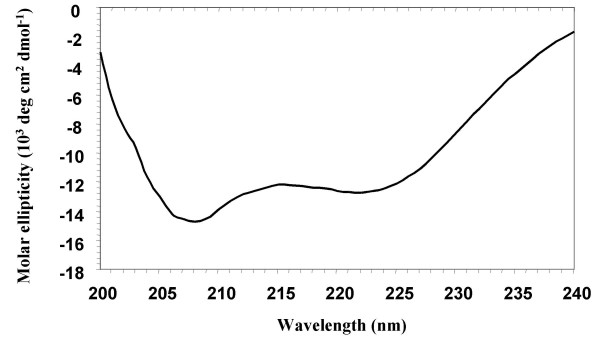
**Evaluation of SSO0551 secondary structure elements by circular dichroïsm**. The molar ellipticity was calculated on the basis of exact amino acid composition of SSO0551 recombinant product.

Content of secondary structure elements in SSO0551 was estimated by far-UV circular dichroïsm. Fig. 3 shows the spectrum recorded at 20°C. Purified protein presents negative ellipticity in the near-UV with minima at 208 (-14.7 10^3 ^deg cm^2 ^dmol^-1^) and 222 nm (-12.7 10^3 ^deg cm^2 ^dmol^-1^). Deconvolution of the CD spectrum leads to an estimation of secondary structural element content of about 28–29 % of α-helices and 14–16 % of β-sheets using K2D neural-software. Predictions of SSO0551 secondary structures by PSIPRED and Jpred web servers gave values of 10–11 % of β-sheets in relative agreement with the circular dichroïsm data, but overestimated the α-helix average content (54 %). PSIPRED and Jpred predictions are based on neural networks trained on known folds. The overestimation of the α-helix content may be due to the novel fold of these COG2042 proteins as discussed here below.

Native molecular mass of SSO0551 was determined by size-exclusion chromatography on a Superdex 200 HR10/30 calibrated column. Pure protein eluted as a peak centered at 39.1 mL in the assay conditions corresponding to an apparent molecular mass lower than 20 kDa. This elution profile indicates that this structured protein behaves as a compact monomer.

### Limited proteolysis defines Glu^73^-Arg^78 ^as a hyper-sensitive region

**Figure 4:**
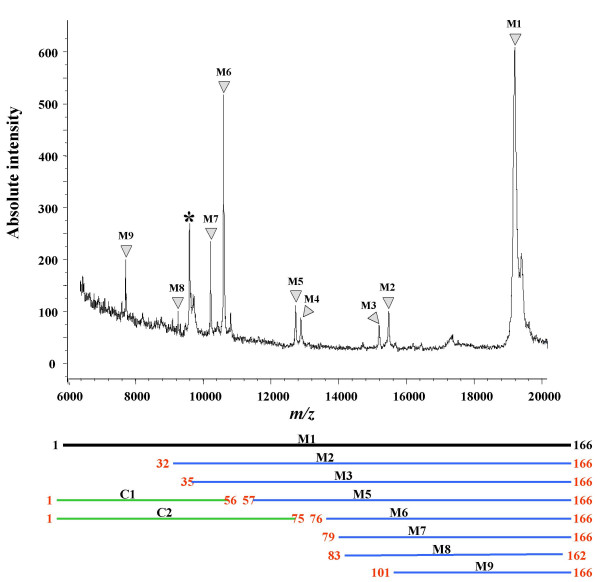
**MALDI-TOF mass spectrum of large peptide fragments produced by SSO0551 partial trypsin proteolysis**. A partial enzymatic proteolysis was carried out during 60 sec at 20°C with a trypsin/SSO0551 protein ratio of 1/20 (w/w). The products were then resolved onto a C8 reverse phase chromatographic column and the different UV absorbing fractions were analyzed by MALDI-TOF. The *m/z *spectrum obtained with the fraction eluting at 40–50 % acetonitrile is shown. The asterisk labels a peak arising from trypsin autolysis. Peaks that could be assigned are identified with numbers (experimental *m/z*, residues, Δmass in ppm compared to theoretical [M+H]^+ ^average mass): **M1 **(19198.4, [1–166], -1), **M2 **(15478.7, [32–166], -53), **M3 **(15191.2, [35–166], +153), **M4 **(12883.4, [52–162] or [56–166], -46 or -43), **M5 **(12724.2, [57–166], +193), **M6 **(10603.8, [76–166], +51), **M7 **(10220.1, [79–166], +77), **M8 **(9257.2, [83–162], +63) and **M9 **(7717.0, [101–166], -134). Peptides **C1 **and **C2**, complementary of **M5 **and **M6**, were not observed in this spectrum but in another fraction from the C8 reverse-phase chromatography corresponding to smaller peptides.

Purified SSO0551 protein was subjected to limited proteolysis with various endopeptidases (trypsin, chymotrypsin, ArgC and GluC). MALDI-TOF mass spectrometry was used to determine cleavage sites by following the time course generation of peptides. Several protease/substrate ratios were assessed to confirm which preferential sites on entire protein were first attacked (earliest cleavage), thus corresponding to a native state of the protein. The two fragments generated by such cleavage may be more vulnerable to subsequent attacks than native protein and therefore late proteolytic sites are considered less informative. Both small and large peptides generated during proteolysis were evaluated. Partial proteolyzed products obtained with trypsin were first resolved by reverse-phase chromatography and analyzed by MALDI-TOF mass spectrometry. Results recorded from direct analysis of the digestions without prior separation were almost similar to those obtained with separation. Therefore, the latter cost-effective strategy was used for analyzing the numerous conditions tested. Figure 4 shows the MALDI mass spectrum of the main large products obtained from a tryptic digest of SSO0551 (enzyme/protein ratio of 1:20) after 60 sec of reaction. In these conditions, the signal of intact protein was still visible at *m/z *19198.4, but mixed with signals corresponding to 8 different large fragments. Among these, 7 peptides arose from an N-terminal proteolysis: [32–166], [35–166], [56–166], [57–166], [76–166], [79–166], [101–166] (Fig. 4). Such peptidic profile indicates that SSO0551 N terminus is rather solvent exposed in comparison to C terminus.

During the earliest events of the trypsin proteolysis analyzed in various conditions for detection of large products but also smaller peptides, monocharged cations with following *m/z*: 8614.6 amu, 10603.4 amu, 6489.8 amu, and 12724.1 amu, were attributed to fragments [1–75] (Δmass: -178 ppm), [76–166] (+89 ppm), [1–56] (+145 ppm), [57–166] (+200 ppm), respectively (data not shown). These data clearly indicate that Lys^75 ^and Arg^56 ^are two sites of early cleavage by trypsin. Identification of peptides Val^32^-Lys^166 ^(15478.7 amu, -53 ppm) and Gly^35^-Lys^166 ^(15191.2 amu, +153 ppm) also indicates that Arg^31 ^and Lys^34 ^could be two other initial nick-sites.

Similar experiments with endoproteinase Arg-C resulted in observation of two pairs of complementary peptides with *m/z *of 1920.9 amu ([1-15] +70 ppm) and 17296.8 amu ([16–166], -95 ppm) on one hand, 8998.1 amu ([1–78], -176 ppm) and 10217.5 amu ([79–166], +332 ppm) on the other hand. These data indicated that Arg^78 ^and Arg^15 ^are the main proteolyzed sites when ArgC enzyme was used. Chymotrypsin attacks SSO0551 native protein mainly at Phe^74 ^because two complementary peptides with *m/z *of 8487.0 amu ([1–74], -249 ppm) and 10734.2 amu ([75–166], -157 ppm) were clearly evidenced. Glu^73 ^is the main proteolyzed site when GluC protease was used, as peptides with *m/z *of 8338.7 amu ([1–73], -118 ppm) and 10880.0 amu ([74–166], +28 ppm) were detected. For all these analysis, smaller peptidic fragments that accumulated over time could be attributed from further proteolysis of the products arising from initial attacks (data not shown). All these results are concordant in pointing out that Glu^73^-Arg^78 ^and Glu^28^-Arg^31 ^are two accessible solvent-exposed regions of the protein as they can be proteolyzed by several endopeptidases, the first cited being definitively hyper-sensitive. Local unfolding not just surface exposure is necessary for efficient *in vitro *proteolysis because the polypeptide segment being cleaved must form a specific structure with the associated protease [30]. For this reason, Glu^73^-Arg^78 ^region should also correspond to a flexible region, i.e. a protruding loop.

### Lysine labeling with NHS-biotin and DTSSP cross-linking confirm that the N terminus is rather solvent-exposed

**Figure 5:**
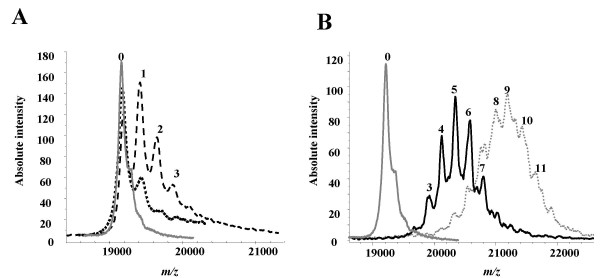
**MALDI-TOF mass spectra of proteins labeled with NHS-biotin**. Protein samples were mixed with various amount of NHS-biotin for 15 min at room temperature. Unlabeled protein mass spectrum is shown in grey solid line. Reagent/total lysine ratios were in Fig. 5A: 1:40 (black dotted line) and 1:20 (black dashed line), and in Fig. 5B: 1:2 (black solid line) and 2:1 (grey dotted line). The number of labeled lysines is indicated above each peak.

The SSO0551 protein contains 21 lysine residues (12 %) distributed along the whole polypeptide sequence. Under mild conditions that should keep the native conformation of the protein, specific labeling of these residues with NHS-biotin may give further details about their respective surface accessibility and/or their interactions with other residues [31]. After reaction with various amount of chemical reagent (molar ratio NHS-Biotin/total lysines of 1:40, 1:20, 1:10, 1:2, 1:1, 2:1), protein labeling was monitored by determining the mass of undigested samples. Figure 5 shows the signals measured by MALDI-TOF mass spectrometry for four of these ratios. The fact that some unmodified protein is still present at ratio below 1:20 testifies for mild conditions that should allow modification of protein still in a native state. As expected with NHS-biotin, each peak exhibits the predictable mass increment (average mass of 226.3 amu per label). Figure 5 shows that at molar ratio of 1:40 a simple modification is obtained, while a more heterogeneous population was detected for higher ratio. For examples, 1 to 3 modifications are detected at ratio 1:20, 2 to 5 modifications at ratio 1:2. However, a limited number of modifications (8–10) are recorded for higher ratio, indicating that among the 21 lysine residues only a fraction is accessible to the chemical.

To localize all labeled residues, NHS-biotin treated samples were subsequently subjected to proteolysis with various endoproteases (trypsin, Arg-C, or Glu-C) and compared to untreated samples. SSO0551 sequence coverage was estimated to be 92 % with all 21 lysine residues included in this coverage. Peptides (Δmass below 120 ppm) detected with NHS-biotin treated samples but not detected with untreated samples are listed in Table 2. Using limiting amount of NHS-biotin (molar ratios of 1:10, 1:20 or 1:40), nine reactive residues are unequivocally identified: Lys^10^, Lys^14^, Lys^20^, Lys^23^, Lys^25^, Lys^51^, Lys^75^, Lys^128^, and Lys^154^, assuming that proteases do not cleave after a modified residue. Other residues, such as Lys^34 ^and Lys^49 ^might be also labeled (Table 2). The number of labeled lysines is in agreement with the limited number of modifications recorded at higher ratio. Remarkably, spectra of whole peptide mixture were informative enough to give assignment of all modified peptides without the need of a purification step. Therefore, other amine reactive reagent that creates a mass shift could have been used.

**Figure 6:**
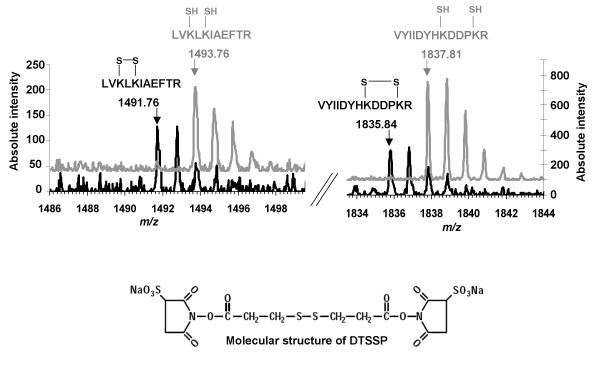
**DTSSP cross-linked peptides identified by MALDI-TOF mass spectrometry**. Protein samples were mixed with DTSSP for 30 min at room temperature at a molar ratio 1:20 (protein:DTSSP). They were then subjected to trypsin proteolysis. Masses of peptides treated with DTT (grey spectrum) or untreated (black spectrum) were measured by MALDI-TOF mass spectrometry. Peptide sequences are indicated and DTSSP cross-links or DTSSP moieties arising from DTT treatment are depicted schematically. Monoisotopic masses of protonated peptides [MH^+^] are theoretically: 1491.807 amu and 1493.822 amu for LVKLKIAEFTR [21-31] cross-linked with one DTSSP molecule (untreated (C_68_H_115_N_16_O_17_S_2_) and DTT-reduced (C_68_H_117_N_16_O_17_S_2_), respectively), 1835.846 amu and 1837.862 amu for VYIIDYHKDDPKR [3-15] cross-linked with one DTSSP molecule (untreated (C_82_H_123_N_20_O_24_S_2_) and DTT-reduced (C_82_H_125_N_20_O_24_S_2_), respectively).

Using a lysine cross-linking reagent, DTSSP, it is possible to assess intra- or inter-molecular protein contacts [21,32]. DTSSP enables cross-linking of amino groups up to 12 Å apart. As SSO0551 was shown to be monomeric and its concentration used in the assays was low (2.5 pM), intramolecular cross-links should be favored over intermolecular cross-links. In addition, the low reagent concentration used should avoid unwanted conformational changes that may be induced by multiple intramolecular cross-linking. After reaction with DTSSP, products were subjected to trypsin proteolysis and peptides were identified by MALDI-TOF. As the protein is relatively small, mass signals could be attributed with a good confidence (tolerance < 120 ppm). In addition, peak attribution was always confirmed upon reduction of products and sometimes through redundancy due to miss-cleavage. SSO0551 sequence coverage was 89%. The monoisotopic cations at *m/z *1169.55, 2077.04, 2715.11 and 2871.14, detected for SSO0551 treated by DTSSP (ratio DTSSP/total polypeptide of 20:1) correspond in mass to addition of a DTSSP moiety on peptides [24-31] (Δmass tolerance: +16 ppm), [123–138] (-7 ppm), [57–78] (+61 ppm) and [56–78] (+82 ppm). Since trypsin does not cleave after a modified lysine, we conclude that Lys^25^, Lys^75 ^and Lys^128 ^were modified. After DTT treatment, peaks corresponding to the expected products (-103.993 amu theoretically) were detected at *m/z *1065.46 (-109 ppm), 1973.03 (0 ppm), 2611.21 (-28 ppm) and 2767.26 (-45 ppm), respectively. Fig. 6 shows two monoisotopic [MH]^+ ^ions at *m/z *1491.76 and 1835.84 corresponding to intrapeptide cross-linked peptides: [21-31] (+31 ppm) and [3-15] (+3 ppm), respectively. These peptides contain two proximal lysine residues (Lys^23^-Lys^25 ^and Lys^10^-Lys^14^). As shown in Fig. 6, these two peaks were absent in mass spectrum following DTT reduction but new peaks at *m/z *1493.69 and 1837.81 appears at the expected increment (+2.016 amu theoretically). An additional peak at *m/z *2502.22 could be attributed to peptide [35–55] (+37 ppm) with an intrapeptide cross-link between Lys^49 ^and Lys^51^. However, the corresponding reduced peak was not detected. Strikingly, every lysines that were reactive with DTSSP were also detected by NHS-biotin labeling. Most modifications are located at the N terminus of the protein where five modified residues belong to the RLI motif.

## Discussion

**Figure 7:**
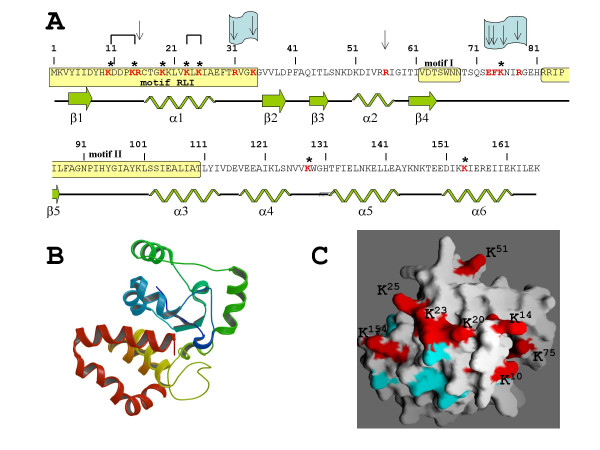
**Experimental topology information obtained on SSO0551 and theoretical 3D model M8 shown to be compatible**. A – Schematic representation of experimental low-resolution structural information. SSO0551 sequence is displayed with its predicted secondary structural elements (α-helices and β-strands are represented by helices and arrows, respectively). The two conserved COG2042 motifs described in this study (I and II) and the 35 amino acids RLI motif are specified. Vertical arrows indicate sites of limited proteolysis that have been experimentally determined. Lysines labeled with NHS-biotin are indicated with asterisks and lysines involved in DTSSP cross-links are schematically bridged. Sequence numbering refers to the native version (untagged) of SSO0551 experimentally characterized. B – Schematic drawing of *ab initio *model M8. Secondary structure elements are shown as coil for α-helices, arrows for β-strands, and lines for loops. The color code follows a rainbow style where N terminus is in blue and C terminus in red. C – Molecular surface of *ab initio *model M8. The localization of solvent-exposed lysines, as determined by NHS-biotin labeling experiments, is colored in red whereas other lysines are colored in blue. This view shows the protein in the same orientation as in B.

Although COG2042 proteins are distributed among a large number of organisms, no experimental evidences have yet been reported concerning their biochemical characterization and function. As they are not related, even remotely, to any other family of proteins, COG2042 members can be phylogenetically considered as orphans. Figure 7 (Panel A) summarizes the structural information obtained with chemical modification approach, in combination with limited proteolysis procedures. Using MALDI-TOF mass spectrometry to identify protease-accessible sites, we have shown that the most exposed regions are located at the first half of the protein, the Glu^73^-Arg^78 ^region being revealed hyper-sensitive to various proteases (Fig. 7A). It probably indicates a protruding loop out of the globular protein. This charged region is relatively conserved among COG2042 orthologs and lies between two highly conserved segments of COG2042 (motif I and II as shown on Fig. 7). Chemical modification agrees with limited proteolysis in that the RLI motif is solvent exposed while the C terminus appeared rather inaccessible (Fig. 7A). The length of the RLI motif, first defined by conserved domain search [33], matches perfectly with two sensitive proteolytic sites (Arg^31 ^and Lys^34^). The RLI domain is also present at the N terminus of another group of orthologous proteins, namely COG1245. Remarkably, COG1245 proteins only occur in two domains of life (Archaea and Eukarya) similarly to COG2042 proteins. Although co-occurrence of protein members is not strictly identical (for example, pyrococci encompass the information for COG1245 but not for COG2042 polypeptides), such occurrence pattern may reflect a functional link between the two protein families.

Our initial objective was to obtain about SSO0551 as much low-resolution structural information as possible in order to discriminate among putative three-dimensional models representing COG2042 protein structure. However, currently available threading tools applied on SSO0551 failed to detect any structurally related-proteins. Alternatively, we obtained ten different *ab initio *models of SSO0551 using the fully-automated ROBETTA server based on ROSETTA procedures [34]. On these ten models, we applied all the low-resolution structural information gathered in this work. We predicted for every model location of preferential proteolytic sites using the NickPred software [35]. Models M1, M2 and M6 on one hand, and M9 and M10 on the other, show hypersensitive regions in the RLI motif or C terminus, respectively. These features do not correspond to our experimental data. Only models M4, M7 and M8 predict that the loop Glu^73^-Arg^78 ^is solvent exposed (data not shown). Among these three models, M4 and M8 respect the ranking of preferential nick-sites for trypsin, chymotrypsin, ArgC and GluC proteases. Solvent accessibility for lysine side chain was evaluated for models M4, M7 and M8 and compared with experimental data (data not shown). All the lysine residues labeled with NHS-biotin are found solvent-exposed in model M8. Manual inspection of cross-linked lysines (Lys^10^-Lys^14 ^and Lys^23^-Lys^25^) revealed that model M4 is not valid because of the opposite orientation of Lys^10 ^and Lys^14^. Figure 7 (Panels B & C) shows cartoon views of the M8 model that fulfills all our experimental constraints. For this model, the distance between the two reactive amine groups of Lys^10^-Lys^14 ^and Lys^23^-Lys^25 ^pairs are 12.7 Å and 13.3 Å, respectively. Search with DALI for structural homologs using model M8 did not result in significant scores with any known PDB structures. This is consistent with the PSI-BLAST results and may indicate that COG2042 proteins share a novel fold. COG2042 proteins are thus a target of choice for genomic structural studies.

In conclusion, we have presented a strategy consisting in obtaining low-resolution structural information (determination of nick-sites, solvent exposed residues, and residue-residue distances) that can be used to distinguish among a large set of theoretical molecular models. Lack of remotely-related structural templates or lack of adequacy between experimental data and most theoretical models indicates that such family of proteins should become a priority in structural genomic projects.

## Methods

### Chemical and biological reagents

Most chemicals used in this study were obtained from Sigma and were of analytical grade. Oligonucleotide primers were purchased from Genset. N-hydroxysuccinimide-biotin (NHS-biotin) and 3,3'-dithio-bis [sulfosuccinimidyl-propionate] (DTSSP) were obtained from Pierce. Matrices for Matrix-assisted Laser Desorption Ionization-Time of Flight (MALDI-TOF) mass spectrometry and calibration standards were purchased from Bruker Daltonics. Sequencing grade proteolytic enzymes were from Roche Applied Science.

### Cloning and overexpression of *SSO0551*

Two constructs were designed in order to get overexpression of the SSO0551 ORF (starting with an ATG codon at nucleotide 484790 on the Crick strand of *S. solfataricus *P2 genome (NC_002754)) and an N-terminal extended version of SSO0551 (starting with an ATG codon at nucleotide 484916). For both proteins, an N-terminal 6His tag was added to render the purification of the recombinant products easier. For this purpose, synthetic oligonucleotide primers were oAB22 (5'-gctagcATGAAGCCCAAACCC-3') and oAB49 (5'-gctagcATGAAGGTATATATTATAGAC-3') that both contain an engineered *Nhe*I site, oAC34 (5'-cggatcctacTCATTTTTCAAGTATTTTC-3') and oAE62 (5'-ggatcctcaTCATTTTTCA AGTATTTTCTC-3') that both contain an engineered *Bam*HI site (restriction sites underlined in the primer sequences and nucleotides not present in the original sequence shown by lower case). Oligonucleotide pairs oAB22/oAC34 and oAB49/oAC34 were used for two distinct PCR amplifications of *SSO0551 *with *S. sulfolobus *total DNA as template. A 643-bp fragment (N-ter 6His-tag extended version of SSO0551) and a 517-bp fragment (N-ter 6His-tag SSO0551) were obtained, respectively. They were cloned into pCRScript-cam (Stratagene), resulting in plasmids pSBTN-AB36 and pSBTN-AB37, respectively. The two inserts were removed by digestion with *Nhe*I and *Bam*HI and ligated with T4 DNA ligase into plasmid pSBTN-AB23 (Armengaud J. & Chaumont V., unpublished data), a derivative of pCR T7/NT-topo (Invitrogen) containing a T7 promoter and 6 His-tag, previously digested with the same endonucleases. The resulting plasmids pSBTN-AB30 and pSBTN-AB31, respectively, were verified by DNA sequencing in order to ascertain the integrity of the nucleotide sequence. Hyperexpression of the recombinant *SSO0551 *constructs was achieved with *E. coli *Rosetta(DE3)pLysS strain (Novagen), freshly transformed with the plasmids described above. Cultures were carried out at 30°C as described earlier [6].

### Purification of recombinant SSO0551 protein

The purification of recombinant SSO0551 was performed from 44 g (wet material) packed cells. Buffer A consisted of 50 mM K_2_HPO_4_/KH_2_PO_4 _buffer (pH 7.2) containing 400 mM K-glutamate. The pellet was thawed on ice and resuspended in 120 mL of buffer A. The cells were disrupted by sonication with a total energy delivered of 71 kJ. The cell-extract was then centrifuged at 30,000 g for 20 min at 4°C to remove cellular debris and aggregated proteins. The supernatant was subjected to a 20 min heat treatment using a water bath maintained at 70°C, and immediately centrifuged a second time at 30,000 g for 20 min at 4°C. Chromatographic steps were performed at room temperature using an Äkta Purifier FPLC system (Amersham Biosciences). The 135 mL supernatant was applied at a flow rate of 2.8 mL/min onto a XK 26 × 20 column (Amersham Biosciences) containing 50 mL of Chelating Sepharose Fast Flow (Amersham Biosciences) and previously loaded with 200 mM NiSO_4_, washed with milliQ water and equilibrated with Buffer A containing 50 mM imidazole. The fraction collected during the IMAC loading was shown to contain the SSO0551 protein. This 222 mL fraction was concentrated to a volume of 56 mL by means of Centricon Plus-20 filtration units (Millipore) and then dialyzed overnight at 4°C against 20 mM K_2_HPO_4_/KH_2_PO_4 _buffer (pH 7.2) containing 20 mM NaCl (buffer B). The 78 mL supernatant obtained after centrifugation at 30,000 g for 10 min at 4°C was divided and applied in two separate runs onto a 6 mLResource-S ion-exchange column (30 mm × 16 mm, 15 μm) from Amersham Biosciences, previously equilibrated with buffer B and operated at a flow rate of 3 mL/min. After a 10 column volume wash with buffer B, proteins were resolved with a 25 column volume linear gradient from 20 to 500 mM NaCl in buffer B. Recombinant SSO0551 was eluted at approximately 250 mM NaCl and desalted by overnight dialysis against Buffer B. The resulting 20 mL protein solution was concentrated to a volume of 8 mL by means of Centricon Plus-20 filtration units (Millipore). The sample was again divided and applied in two separate runs onto a superdex75 gel filtration packed into a HR 16/50 column at a flow rate of 1.5 mL/min in 20 mM K_2_HPO_4_/KH_2_PO_4 _buffer (pH 7.2) containing 100 mM NaCl. The fractions obtained with the two runs were pooled and dialyzed overnight at 4°C against 10 mM HEPES buffer (pH 7.2). After dialysis, the fraction was centrifuged at 26,000 g for 20 min at 4°C and the protein concentration was measured by spectrophotometry using a molar absorption coefficient of 19060 M^-1 ^cm^-1 ^at 280 nm. The purified protein was flash frozen in liquid nitrogen and stored at -80°C at a concentration of 0.48 mg/mL.

### Circular dichroïsm

Far- and near-UV circular dichroism spectra were recorded at 20°C between 200 and 300 nm on a J-810 Jasco spectropolarimeter equipped with a PTC-424S Jasco Peltier, using a quartz cuvette of 1 mm path length, with a 20 nm/min scanning speed and a band-width of 1 nm. Three spectra of purified SSO0551 at 1.92 μM in 10 mM HEPES buffer (pH 7.2) were averaged and corrected from the baseline for buffer solvent contribution. Experimental data were analyzed using the program K2D [36] described by Andrade et al. [37].

### Determination of native molecular mass by gel filtration

The native molecular mass of SSO0551 was estimated by gel filtration chromatography on a Superdex 200 gel packed into a HR10/30 column (Amersham Biosciences) with a final bed volume of 24 mL. The column was equilibrated at room temperature at a flow rate of 0.5 mL/min with 50 mM Tris/HCl buffer, pH 8.3, containing 50 mM NaCl and eluted with the same buffer. Protein standards used to calibrate the column were ribonuclease A (15.8 kDa), chymotrypsinogen A (21.2 kDa), ovalbumin (49.4 kDa), albumin (69.8 kDa), aldolase (191 kDa) and catalase (215 kDa), all from Amersham Biosciences. Exclusion limit was evaluated with dextran blue 2000 (Amersham Biosciences). A sample consisting of 90 μL of SSO0551 at 25.2 μM was injected and specific absorptions at 280 and 266 nm were followed.

### Mass spectrometry

Matrix-Assisted Laser Desorption/Ionization Time-Of-Flight (MALDI-TOF) mass measurements were performed using a Biflex IV instrument (Bruker Daltonics) in positive ionization mode. Protein samples and large peptidic fragments (>3500 Da) were applied to the target using sinapinic acid prepared as saturated solution in 30 % acetonitrile, 70 % milli-Q water and 0.1 % TFA as matrix. Samples were prepared using the dried droplet method and measured in linear mode. Small peptide samples were measured in reflectron mode using α-cyano-4-hydroxycinnamic acid in 30% acetonitrile containing 0.1% trifluoroacetic acid as matrix. Mass spectra were obtained by summation of 100–210 laser shots. The instrument was calibrated for determination of entire protein masses using either a mixture of chymotrypsin and bovine serum albumine, or apomyoglobin and aldolase. For peptides, the instrument was calibrated using a pepmix calibration kit (Bruker Daltonics). When necessary, the mass spectrometer was also internally calibrated using some of the theoretical peptide masses.

### Limited protease digestion

For *in-solution *partial digestion, 0.2 nmol of pure SSO0551 were diluted into buffer D1 (20 mM TRIS/HCl, pH 7.8), buffer D2 (20 mM NH_4_HCO_3_, pH 7.8) or buffer D3 (20 mM TRIS/HCl, pH 7.8, containing 10 mM CaCl_2 _and 5 mM DTT). Trypsin or chymotrypsin was added to SSO0551 diluted into buffer D1, whereas Glu-C or Arg-C was added to the protein diluted into buffer D2 or D3, respectively. Several enzyme/protein ratios (1:50 (w/w), 1:20 (w/w) and 1:2 (w/w)) were tested for each endoprotease. The digestions were performed at room temperature and aliquots were analyzed from 30 sec to 10–240 min. Digested samples were desalted using ZipTip_C18 _or ZipTip_C4 _pipette tips (Millipore) according to the protocol specified by the manufacturer and their mass directly evaluated by MALDI-TOF. Eventually, partially proteolyzed mixtures of larger quantities (10 nmol of SSO0551) were fractionated by reverse-phase HPLC using an Aquapore RP-300 column (PerkinElmer; 100 × 1.0 mm, 7 μm, 300 Å pore size) developed at 200 μL/min with a linear gradient from 5 to 90 % of acetonitrile in TFA 0.1 % over 45 min. The elution was monitored at 220 nm with an Agilent 1100 Series HPLC system equipped with a G1315 diode array detector. Individual fractions were concentrated by evaporation in a SpeedVac (Savant) and directly analyzed by MALDI-TOF.

### Lysine labeling by NHS-biotin

N-hydroxysuccinimide-biotin (NHS-biotin) was used to label ε-amino groups of SSO0551 lysines. After reaction the biotin labels resulted coupled to the lysines through a stable amide bond. The increase in mass for each label (C_10_H_14_N_2_O_2_S_1_) should be 226.293 amu if average mass is considered or 226.078 amu in monoisotopic mode. Modification of lysine residues was carried out by incubating 1.25 nmol of SSO0551 in 20 mM HEPES, pH 7.2, with various amount of freshly prepared NHS-biotin reagent dissolved in anhydrous dimethylsulfoxide. After 30 min of incubation at room temperature, the reagent in excess was removed by a 30 min micro-dialysis against 20 mM HEPES, pH 7.2. Samples were directly desalted by using ZipTip_C4 _(Millipore) prior MALDI-TOF analysis. They were eventually digested overnight with an endoprotease (trypsin, GluC or ArgC) and desalted by using ZipTip_C18 _pipette tips (Millipore) prior mass analysis.

### Lysine cross-linking with DTSSP

3,3'-Dithio-bis [sulfosuccinimidyl-propionate] (DTSSP) was used to cross-link two ε-amino groups of SSO0551 lysines, essentially as described in [32]. The mass increase (in monoisotopic mode) for each label should be 191.991 amu (C_6_H_8_O_3_S_2_) or 87.998 amu (C_3_H_4_O_1_S_1_) when DTT treated. The increase in mass for an intramolecular cross-link between two lysines should be 173.981 amu (C_6_H_6_O_2_S_2_) or 175.997 amu (2 × C_3_H_4_O_1_S_1_) when DTT treated. Therefore after reduction of the disulfide bridge by DTT, an additional increase of 2.016 amu should be measured. Reaction was carried out by incubating 0.25 nmol of SSO0551 in 20 mM NaH_2_PO_4_/Na_2_HPO_4_, pH 7.5 containing 150 mM NaCl, with various amount of DTSSP reagent (molar ratio of 20, 35, and 50 mol of DTSSP per mol of polypeptide). After 30 min of incubation at room temperature, the reagent in excess was removed by a 30 min micro-dialysis against 20 mM NaH_2_PO_4_/Na_2_HPO_4_, pH 7.5 containing 150 mM NaCl. Prior overnight trypsin proteolysis, urea (330 mM final concentration) was added to each sample. Before being desalted by using ZipTip_C18 _pipette tips (Millipore), the digested peptide mixture was eventually reduced with 50 mM DTT for 30 minutes at 37°C to reduce the thiol linker.

### *In silico *analysis

Sequence searching was performed using PSI-BLAST with default parameters. Multiple sequence alignments were performed using VectorNTI software package (Informax Inc). Secondary structure predictions were obtained through the PSIPRED v2.4 web-interfaced facilities [38] described by McGuffin et al. [39]. The molar absorption coefficient at 280 nm for SSO0551 was obtained from calculation of the amino acid composition of the recombinant protein [40,41]. Isotopic and average mass of both DTSSP cross-linker and NHS-biotin were calculated using a web-interfaced molecular weight calculator [42]. The peptide assignment and the first attempt for identifying the labeled products and cross-linking products were performed using the FindMod package at ExPaSy [43]. If no match was found, a more detailed search for multiple labels or combinatorial cross-linkable peptide pairs was carried out. Partially proteolyzed products were assigned using the FindPept tool [44]. Tertiary structure predictions were carried out using publicly available online services, including 3D-PSSM [45], FUGUE [46] and PSIPRED [39]. *Ab initio *modeling was performed using the ROBETTA server [34,47]. Each model was analyzed in terms of proteolytic sensitivity using the NICKPRED software [35,48,49]. Residues accessibility have been calculated using a modified version of Connolly's MS program ([50]; Pellequer JL, unpublished results). Structural homologs were searched using DALI web server from the European Bioinformatics Institute [51]. Model views were obtained with the MOLSCRIPT program [52] and rendered using RASTER3D [53].

## List of abbreviations

**amu**, atomic mass unit; **COG**, Cluster of Orthologous Group; **DTSSP**, 3,3'-dithio-bis [sulfosuccinimidyl-propionate]; **IPTG**, isopropyl-γ-D-thiogalactopyranoside; **HPLC**, high performance liquid chromatography; **EDTA**, Ethylenediaminetetraacetic acid; **HEPES**, 4-(2-hydroxyethyl)piperazine-1-ethanesulfonic acid; **HEPPS**, N-(2-hydroxyethyl)piperazine-N'-(3-propanesulfonic acid);**IMAC**, immobilized metal ion adsorption chromatography; **MALDI-TOF**, Matrix-assisted Laser Desorption/Ionization Time-of-Flight; **NHS-biotin**, N-hydroxysuccinimide-biotin; **PSI-BLAST**, Position-Specific Iterated Blast; **Tris**, 2-[bis(2-hydroxyethyl)amino]-2-(hydroxymethyl)-propane-1,3-diol.

## Authors' contributions

JA conceived, coordinated the study and participated in all its experimental aspects. He analyzed the genomic distribution of this family of proteins, designed and engineered the recombinant SSO0551 molecule, conceived the mass spectrometry strategies and interpreted the data. He proposed *ab initio *modeling of SSO0551 and drafted the original manuscript. AD actively participated in conception of the mass spectrometry strategies, advised OS on execution and interpretation of mass spectrometry experiments and assisted in figure design. OS performed and interpreted all mass spectrometry experiments. JLP contributed its experience for the modeling aspects of the project. EQ contributed its experience in mass spectrometry-based topology. All authors participated in manuscript preparation, read and approved the final manuscript.

## Figures and Tables

**Table 1:** Fingerprint identification of recombinant products from pSBTN-AB31 and pSBTN-AB30 constructs.

**pSBTN-AB31 construct**^a^		**pSBTN-AB30 construct**^a^		**Theoretical peptides**		
[MH]^+ ^observed (in amu)	Δmass (in ppm)	[MH]^+ ^observed (in amu)	Δmass (in ppm)	[MH]^+ ^expected (in amu)	Position^b ^(Start-End)	Peptide assignment

1590.64	-31	nd ^c^	-	1590.69	3–16	GSHHHHHHGMASMK
nd ^c^	-	1050.54	-19	1050.56	17–24	VYIIDYHK
1505.71	-33	nd ^c^	-	1505.76	17–28	VYIIDYHKDDPK
1661.83	-18	1661.85	-6	1661.86	17–29	VYIIDYHKDDPKR
1601.84	-31	1601.83	-37	1601.89	49–63	GVVLDPFAQITLSNK
1844.98	-16	1845.00	-5	1845.01	49–65	GVVLDPFAQITLSNKD
2328.28	-4	2328.28	-4	2328.29	49–69	GVVLDPFA...KDKDIVR
2484.35	-16	2484.34	-20	2484.39	49–70	GVVLDPFA...KDKDIVRR
2140.06	0	2140.06	0	2140.06	71–89	IGITIVDTSWNNTSQSEFK
2296.16	0	2296.16	0	2296.16	70–89	RIGITIVDTSWNNTSQSEFK
2043.17	10	2043.18	15	2043.15	97–114	RIPILFAGNPIHYGIAYK
1887.05	0	1887.06	5	1887.05	98–114	IPILFAGNPIHYGIAYK
1244.62	-16	1244.59	-40	1244.64	143–152	WGHTFIELNK
2091.09	0	nd ^c^	-	2091.09	143–159	WGHTFIELNKELLEAYK
nd ^c^	-	865.53	69	865.47	153–159	ELLEAYK

^a^The two products were isolated from SDS-PAGE and treated with trypsin. The resulting peptides were analyzed by MALDI-TOF spectrometry in reflectron mode. The expected length for the two products are: 180 amino acids for SSO0551 with the 6His-tag at the N terminus (pSBTN-AB31 construct) and 222 amino acids for SSO0551 extended version (42 additional amino acids at the N terminus and a 6His-tag; pSBTN-AB30 construct).

^b^Peptide positions refers to SSO0551.

^c^«nd» denote the peptides not detected in one of the samples.

**Table 2:** Monoisotopic [M+H]^+ ^peptides generated by various proteases after NHS-biotin labeling of SSO0551.

Protease	[M+H]^+ ^(observed)	[M+H]^+ ^(expected)	Δmass (ppm)	Position (Start-End)	Peptide assignment^a^	NHS-biotin label^b^	Modified residues^a^
Trypsin	1731.94	1731.84	-57	3–14	VYIIDYH**K**DDPK	1	**K^10^**
Trypsin	2114.27	2114.02	118	3–15	VYIIDYH**K**DDP**K**R	2	**K^10^**and**K^14^**
Trypsin	1888.15	1887.94	111	3–15	VYIIDYHKDDPKR	1	K^10^orK^14^
Trypsin	1991.21	1990.98	-119	1–14	MKVYIIDYHKDDPK	1	K^2^orK^10^
Trypsin	2147.33	2147.08	-120	1–15	MKVYIIDYHKDDPKR	1	K^2^orK^10^orK^14^
Trypsin	2163.33	2163.07	-120	1–15	M^oxy^KVYIIDYHKDDPKR	1	K^2^orK^10^orK^14^
Trypsin	2373.41	2373.16	-107	1–15	MKVYIIDYHKDDPKR	2	K^2^or/andK^10^or/andK^14^
							
Trypsin	1180.77	1180.69	-64	20–25	**K**LV**K**LK	2	**K^20^**and**K^23^**
Trypsin	1203.76	1203.66	-87	24–31	L**K**IAEFTR	1	**K^25^**
Trypsin	1770.16	1769.98	102	21–31	LV**K**L**K**IAEFTR	2	**K^23^**and**K^25^**
Trypsin	1898.15	1898.08	-39	20–31	KLVKLKIAEFTR	2	K^20^orK^23^orK^25^
							
Trypsin	1127.68	1127.60	-71	50–56	D**K**DIVRR	1	**K^51^**
Trypsin	3064.63	3064.63	1	32–55	VGKGVVL...NKDKDIVR	2	K^34^or/andK^49^or/andK^51^
Trypsin	2554.57	2554.37	-78	35–55	GVVL...NKDKDIVR	1	K^49^orK^51^
Trypsin	2710.61	2710.47	-51	35–56	GVVL...NKDKDIVRR	1	K^49^orK^51^
Trypsin	2838.65	2838.55	-34	32–55	VGKGVVL...NKDKDIVR	1	K^34^orK^49^orK^51^
Arg-C	2838.75	2838.55	-69	32–55	VGKGVVL...NKDKDIVR	1	K^34^orK^49^orK^51^
							
Trypsin	2749.36	2749.36	0	57–78	IGIT...SEF**K**NIR	1	**K^75^**
Arg-C	2749.60	2749.36	-87	57–78	IGIT...SEF**K**NIR	1	**K^75^**
Glu-C	1089.56	1089.55	-8	74–80	F**K**NIRGE	1	**K^75^**
							
Trypsin	2111.22	2111.11	-52	123–138	LSNVVKWGHTFIELNK	1	**K^128^**
Glu-C	2068.19	2068.10	-41	120–135	AIKLSNVVKWGHTFIE	1	K^122^orK^128^
							
Trypsin	1330.74	1330.67	-55	146–154	NKTEEDIKK	1	K^147^orK^153^
Glu-C	1256.65	1256.67	13	151–158	DIKKIERE	1	K^153^orK^154^
Trypsin	1486.85	1486.76	-60	148–157	TEEDIKKIEREIIEK	1	K^153^orK^154^
Trypsin	1383.76	1383.77	5	154–162	**K**IEREIIEK	1	**K^154^**
Trypsin	1867.14	1867.07	-36	154–166	KIEREIIEKILEK	1	K^162^orK^154^
Trypsin	1340.71	1340.75	29	158–166	EIIEKILEK	1	K^162^orK^166^
Trypsin	1739.01	1738.98	-19	155–166	IEREIIEKILEK	1	K^162^orK^166^

^a^Unequivocal modified residues are indicated in bold characters.

^b^NHS-biotin label (+226.08 amu).
